# Children and Nature: Linking Accessibility of Natural Environments and Children’s Health-Related Quality of Life

**DOI:** 10.3390/ijerph15061072

**Published:** 2018-05-25

**Authors:** Suzanne Tillmann, Andrew F. Clark, Jason A. Gilliland

**Affiliations:** 1 Human Environments Analysis Laboratory, Department of Geography, University of Western Ontario, London, ON N6A 3K7, Canada; stillma@uwo.ca (S.T.); aclark2@uwo.ca (A.F.C.); 2 Department of Paediatrics, University of Western Ontario, London, ON N6A 3K7, Canada; 3 School of Health Studies, University of Western Ontario, London, ON N6A 3K7, Canada; 4 Department of Epidemiology and Biostatistics, University of Western Ontario, London, ON N6A 3K7, Canada; 5 Children’s Health Research Institute, London, N6C 2V5, Canada; 6 Lawson Health Research Institute, London, N6C 2R5, Canada

**Keywords:** children, mental health, health-related quality of life, nature

## Abstract

A growing body of research suggests that increasing children’s nature interactions can have positive benefits for their health-related quality of life (HRQOL); however, researchers have yet to examine how geographical context influences this relationship. The purpose of this study was to examine individual-level and environmental factors that are associated with HRQOL of children from different geographical contexts. Data were collected for 851 children from 34 elementary schools in Ontario, Canada. The natural environments around each child’s home were computed using geospatial analyses in a geographic information system. Natural environment measures were combined with HRQOL and the demographics from child surveys to be used in a series of step-wise linear regression models. These models explored the relationship between children’s HRQOL and the natural environment in urban/suburban and rural populations. In addition to important individual-level determinants, the findings revealed that characteristics of the natural environment, including the amount of greenness, park, and water, show significant relationships in the urban/suburban population. Interpersonal variables were the key predictors of HRQOL in the rural population. Where children live influences relationships between nature and HRQOL. These findings have implications for policymakers, health practitioners, educators, and parents in the design and the promotion of nature for children’s HRQOL.

## 1. Introduction

A growing body of research, especially over the last decade, has focused on the relationship between nature and children’s physical, mental, social, and cognitive health and development [1,2,3,4,5]. This research is vital for the development of new strategies for improving children’s overall health and well-being. This paper examines how factors at the intrapersonal, interpersonal, and physical environment level can influence this relationship. 

Health-related quality of life (HRQOL) is a common measure that is used to assess the distinct aspects of quality of life, including a child’s physical, emotional, social, and school functioning [6,7]; thus, providing a greater level of detail than general quality of life [8,9]. These measurements of HRQOL are subjective and multidimensional as they represent the personal perception of a participant and include a broad range of health and life outcomes [10]. A limited number of studies have also used HRQOL as a measure to assess mental health [7,11,12]. A child’s ability to easily and properly function physically, emotionally, socially, and in-school can all be factors that influence mental health outcomes, such as self-esteem, anxiety, depression, ADD/ADHD, and resiliency. HRQOL allows for the opportunity to easily assess various aspects of well-being that can contribute to the overall mental health of a child. While many authors consider mental health a measure of the absence or presence of a clinical illness or disorder (e.g., bi-polar disorder or schizophrenia), we adopt the WHO definition of mental health as “a state of well-being in which every individual realizes his or her own potential, can cope with the normal stresses of life, can work productively and fruitfully, and is able to make a contribution to her or his community” [13].

The multidimensional aspect of this measurement helps to explain more than one aspect of children’s health, as a large majority of research measuring the potential benefits of nature on children focuses directly on one measure of health, such as physical activity [14,15]. However, the growing body of literature and public opinion on the benefits of nature for children’s health excludes substantial evidence on the relationship between children’s HRQOL and nature. A recent systematic review identified only two papers that have investigated the link between nature and HRQOL in children, with inconclusive results [16]. Kim et al. [11] found that larger and more tree areas in the neighbourhood was associated with the likelihood of having a higher HRQOL in children aged 9–11. Where, McCracken et al. [12] found that there was no significant effect of the quantity of residential green space and HRQOL in children aged 8–11. 

Nature is commonly acknowledged as being beneficial to human health [17,18,19,20,21]; however, the type, dose, and duration that are associated with nature’s impact on health are largely still to be confirmed [19]. How nature and interactions with nature are operationalized are major factors in considering the effects of the potential benefits of nature on children’s health. Previous research has conceptualized three general types of nature interaction: accessibility, exposure, and engagement [16]. The term accessibility refers to whether or not specific element(s) of nature exist within a child’s environment, usually within a pre-defined meaningful distance [22,23,24] (e.g., walking distance from home), and it is often quantified as a given amount or density of the natural element(s). The term exposure is used to refer to situations where there is a direct encounter or contact with nature, and it is usually operationalized in terms of time that is spent in or near, or simply the use of, a natural area. Engagement is a third general type of nature interaction, which refers to the intentional interaction with a natural environment with the purpose of being in nature, such as wilderness therapy, gardening, or outdoor adventure camps. It is necessary for researchers to distinguish the types of nature interaction if we are to determine which can be the most influential in benefiting children’s health. 

Understanding the health disparities that exist between urban and rural environments is important for building and sustaining effective policy and programs for healthy lifestyles [25]. Some previous research has focused on urban children or urban spaces and HRQOL [6,12]; however, there is little evidence on rural [26] and suburban children’s HRQOL. Researchers agree that geographical context, including the level of urbanicity, can modify the health outcomes that are associated with green space exposure [27]. Studies measuring urban green space or rural green space have come to similar conclusions generally, pointing to a positive relationship with health; however, the majority of these studies do not compare urban and rural, and even fewer studies specifically consider suburban environments.

This study builds upon existing literature exploring the impact of accessibility to nature on children’s well-being. More specifically, this study aims to fill gaps in the current literature by investigating the relationship between nature and HRQOL among children that are living in a variety of geographical contexts across the province of Ontario in Canada. Ontario offers a wide range of geographical environments, as it spans over seven degrees latitude. Therefore, this study had two key research objectives: (1) to evaluate the effect of accessibility to natural environments on children’s HRQOL with respect to four domains (physical, emotional, social, and school); and, (2) to analyze how this relationship varies for children living in different geographical contexts (i.e., urban, suburban, and rural; Northwestern versus Southwestern Ontario). 

## 2. Materials and Methods

This study draws from an ongoing research project (2010–2016), which is called the Spatial Temporal Environment Activity Monitoring (STEAM) Project, which examines the environmental influences on health-related behaviours and outcomes among children aged 8 to 14 years (further details can be found elsewhere [28,29]). It was approved by the Non-Medical Research Ethics Board of the University of Western Ontario (STEAM South NM-REB #:17918S; STEAM North NM-REB #:108029) and all respective research officers/committees of the participating school boards. All children in grades five through eight in 34 participating schools were eligible, provided that they obtained signed parental consent and gave child assent. The sample from Northwestern Ontario also included grade four students from three of the participating schools.

For the purposes of this paper, the demographic surveys, PedsQL responses, and GPS data logs were used in combination to assess how accessibility to nature around the home affects children’s HRQOL. All six indices that can be calculated using the validated tool for children aged 8–12 years exceeded the recommended minimum alpha coefficient standard of 0.70 for group comparisons [30].

The study participants come from the first phase of each round of the STEAM Project that included the PedsQL questionnaire during the fall season of data collection years 2011–2013 and 2016 (*n* = 926). Participant data was not eligible for analysis if the PedsQL was missing more than 50% of the items within each scale or if a home location was unable to be determined from each child’s GPS data. The final sample of participants includes 851 children. Schools in the study were distributed across the urban, suburban, and rural environments, with four from Northwestern Ontario and 30 from Southwestern Ontario. As the 34 schools are located in diverse environments, children have differing levels of access to a wide range of natural features (e.g., public parks deigned and maintained by municipalities, environmentally protected areas, forests, rivers, lakes, open spaces). Descriptive statistics about the participants can be found in Results.

### 2.1. Measures

#### 2.1.1. Dependent Variable

The dependent variables for this analysis include HRQOL and its associated domains of children’s health, as measured by the Pediatric Quality of Life Inventory 4.0 (PedsQL) [30,31,32,33]. The PedsQL is a popular tool in children’s health research, which is a modular approach to measuring the HRQOL, and as a result, mental health, in both healthy and ill child/adolescent populations (http://www.pedsql.org/). The child self-report is designed to measure HRQOL in children ages 8–12. It can be broken down into four domains of HRQOL: Physical Functioning (eight items), Emotional Functioning (five items), Social Functioning (five items), and School Functioning (five items). Each domain asks how much of a problem each item has been in the last month: never, almost never, sometimes, often, or almost always. Each item is reversed scored to transform the raw score (0–4) to a value out of 100: Never (0, 100), Almost Never (1, 75), Sometimes (2, 50), Often (3, 25), and Almost Always (4, 0).

#### 2.1.2. Independent Variables

Using the socio-ecological model of health [34], this study identifies three groups of independent variables: intrapersonal, interpersonal, and physical environment.

Intrapersonal variables were collected from the youth surveys. The models described in this study were measured for each individual child, and include: gender (girl vs. boy), age, and visible minority (white/Caucasian vs. non-white/Caucasian).

Interpersonal variables were collected from youth and parent surveys. The models that are described in this study were measured for each individual child and include: lone parent household, live in more than one home, the presence of siblings, parental education status, parental employment status, and household income. 

Physical environment variables are measured based on a child’s exact home location, as identified by passive GPS tracking. Physical environment measures based on accessibility around a child’s home were calculated in ArcGIS v10.4 (ESRI, Redlands, CA, USA) [35]. Accessibility to nature was defined using Euclidean buffers at 500M that were generated using GIS. Many studies using buffers to assess physical activity in an individual’s environment discuss how appropriate buffer sizes largely depend on the environmental context, behaviour of interest, and the group being studied [36,37]. The buffer size chosen for this study was based on those used in previous studies that were exploring children’s neighbourhood environments [22,23,24]. Euclidean buffers were used instead of network buffers, as the natural environment was the main independent variable of interest. Network buffers often eliminate green space due to the nature of their design in that they are shaped by the configuration of the street network, which is less relevant to the way that children move through nature [38]. As Clark, Bent, and Gilliland [39] have previously demonstrated using GIS and field observations, children often use shortcuts (e.g., bike paths, parks, fields, multiuse pathways) to move through their environments, rather than restricting their movement to the traditional roadways.

Using land use data for park and water variables from DMTI Spatial Inc., (Richmond Hill, ON, Canada) park layers from corresponding cities and counties, and water layers from Natural Resources Canada CanVec, we measured the ratio of parks and water to total area of each 500M buffer for each child. The two variables that were developed include: *Park* and *Water*, where each measure was defined as the percentage of each feature within the buffer area.

Normalized Difference Vegetation Index (NDVI) is commonly used as a means of measuring greenness for spatial epidemiologic purposes using satellite imagery [40]. For this study, all of the images were extracted from dates, according to the corresponding study period, Landsat 8 images [41], for 2012–16 participants and Landsat TM images (2011) was used for 2011 participants. Our final measures using NDVI includes grass and shrubbery (NDVI values of 0.2–0.6), and dense vegetation (NDVI values ≥ 0.6) [42]. NDVI variables were calculated from 30 m resolution images, where each value within the buffer was aggregated to calculate the percentage of area within a buffer that each of the two NDVI categories covered. All of the images were extracted during the summer months to maximize green coverage in study areas.

Physical environment measures that were based on home location include geographical context and region of Ontario. The geographical context was defined by three levels of urbanicity, including urban, suburban, and rural. Each level was defined based on urban morphology and the population of included cities and towns. Urban and suburban populations were included in one analysis that controlled for urbanicity (urban versus suburban). Region of Ontario was based on the study region where each participant came from (Northwestern vs. Southwestern). This variable was only included in the rural population analysis, as no urban or suburban population existed in the Northwestern Ontario study area. 

### 2.2. Statistical Analysis

Statistical analysis was performed with IBM SPSS Statistics 24 and STATA SE 13 64 bit (SPSS, Armonk, NY, USA; STATA College Station, TX, USA) [43,44]. Step-wise linear regression [45] was used to develop predictive models of HRQOL and the related indices (i.e., total, psychosocial, physical, emotional, social, school) for different geographic contexts: (1) urban/suburban environments; and, (2) rural environments. Independent variables were introduced into the models step-wise based on the socio-ecological model, including intrapersonal, interpersonal, and physical environmental factors. The level of significance used for analysis was *p* < 0.05.

To better contextualize the results of the models, the resulting coefficients are compared to a minimal clinically meaningful difference (CMD) in scale scores for each indicator of HRQOL using a standard error of measurement [30]. The standard error of measurement was calculated for each of the HRQOL indices of the current study by multiplying the standard deviation by the square root of 1-alpha of each index (Cronbach alpha reliability coefficient) (See Table 1). These important differences will be used in discussing the findings of the current study.

## 3. Results

### 3.1. Descriptive Statistics

Descriptive statistics about the sample can be found in Table 2. The majority of participants were between ages 11 and 12 (70.7%). Of the participants, 55.5% were girls and 44.5% were boys. The majority came from a two-parent household (69.3%) and identified as white (69%). Only 15.9% of the sample lived in more than one home, where 30.6% of participants lived in a lone parent household. Mothers having some post-secondary education and employment were identical values at 61.6%. Whereas, 67.9% of fathers were employed and only 52.3% had some post-secondary education.

The average percentage of park space within the 500M buffer around participant’s homes is 7.4% (urban/suburban: 11.4%, rural: 2.5%), whereas water space was only 1.5% (urban/suburban: 1.0%, rural: 2.0%). The percentage of grass and shrubbery within the 500M buffer of home was higher than the dense vegetation index, 56.2% versus 38.4% (urban/suburban: 67.8% versus 42.1%, rural: 26.2% versus 53.3%). The majority of participants lived in suburban (45.6%) or rural areas (45.1%) and in Southwestern Ontario (84.8%). HRQOL scores show the mean value for each index.

### 3.2. Model Results

The results for only the 500M buffer were presented here, as this buffer size explained the most variance, with similar variables showing significance at additional buffer sizes (see Supplementary Tables S1–S6). The model fit (R^2^) increases as each level of variables were added. The addition of interpersonal values to the model saw the greatest change in R^2^, while there was an increase in explained variance at each step; however, some were greater than others. The results of the final models from the step-wise regression were shown for each of the six dependent variables for both the urban/suburban and rural populations. Results from each of the models were displayed in Table 3 and Table 4. Only variables that were found to be significant when *p* < 0.05 at the 500M buffer were discussed here.

#### 3.2.1. Urban and Suburban Population

The results (Table 3) show that no intrapersonal or interpersonal level variables were predictors for total scale scores. The percentage of water and grass and shrubbery index of NDVI were both negatively statistically significant at the 99% and 95% confidence level, respectively. Percentage of park space was positively associated with total scale scores at the 95% confidence level. Results from psychosocial health show that no intrapersonal or interpersonal level variables were predictors. Percentage of water and grass and shrubbery were negative significant predictors at the 99%, and 95% confidence level, respectively. No intrapersonal or interpersonal variables were predictors in the model for physical functioning. Three natural environment variables were significant predictors of physical functioning. Percentage of park space was the only positive predictor, at the 95% confidence level. The percentage of water and grass and shrubbery, were negatively associated with physical functioning, at the 99% confidence level.

Water was the only statistically significant predictor of emotional functioning, demonstrating a negative association at the 99% confidence level.

There were no intrapersonal variables that were significant predictors of social functioning. The presence of siblings shows a positive association with social function, at the 95% confidence level. Percentage of park, water, and grass and shrubbery were all significant predictors at the 95%, 99%, and 95% confidence level. Parks were the only positive predictor.

Results from examining school functioning show that the interpersonal variable living in more than one home (β = 5.74) is a significant predictor, at the 95% confidence level, and this was the only variable to show a clinically meaningful difference for the urban/suburban population (CMD > 4.79). The percentage of grass and shrubbery was negatively associated with school functioning, at the 99% confidence level.

#### 3.2.2. Rural Population

The results of the models (Table 4) for the rural population of the sample overall demonstrate no significant association between the physical environment variables and HRQOL scores. Results from the total scale score model show that high household income was the only positive predictor at the 95% confidence level.

Psychosocial health scores were positively predicted by age and high household income, at the 95% and 99% confidence level, respectively.

Only one interpersonal level variable was a significant predictor of physical functioning, where living in one home was a negative significant predictor, at the 95% confidence level.

Gender and medium household income were both significant positive predictors of emotional functioning, at the 99% and 95% confidence level, respectively. Boys have significantly higher scores than girls.

Social functioning scores saw all three levels of variables to be significant predictors. Age was a positive predictor at the 99% confidence level. Medium and high household income were both positive predictors at the 95% and 99% confidence level, respectively. Both measures of NDVI, grass, and shrubbery and dense vegetation, were positive predictors, at the 95% confidence level.

Results from examining school functioning scores demonstrate high household income to be the only positive predictor, at the 95% confidence level.

The relationship between gender (β = 6.77) and emotional functioning was the only intrapersonal predictor to show clinically important differences (CMD > 5.83). Two interpersonal variables demonstrate a clinically meaningful difference in the rural population, living in more than one home and household income. Living in more than one home (β = −4.48) was clinically meaningful for physical functioning (CMD > 3.89). Medium household income was clinically meaningful in both emotional (β = 6.78, CMD > 5.83) and social functioning (β = 6.90, CMD > 5.46), whereas high household income was clinically meaningful in total scale scores (β = 5.87, CMD > 4.57), psychosocial health (β = 7.25, CMD > 5.29), social (β = 11.11, CMD > 5.46), and school functioning (β = 5.96, CMD > 4.79). 

## 4. Discussion

This study examined whether factors at multiple levels of the socio-ecological model predict children’s HRQOL. Intrapersonal, interpersonal, and physical environment variables were used to predict HRQOL. Park, water, and NDVI measures at a 500M buffer around the home also proved to demonstrate a significant relationship in predicting certain HRQOL scores. Differences exist between the urban/suburban and the rural populations that were investigated in the current study. However, many of these predictors are not seen as clinically meaningful.

Intrapersonal variables were only significant predictors in the rural population. For girls, emotional functioning was associated with lower scores, while also being associated with higher social and school functioning scores. However, the relationship between gender and emotional functioning in the rural analysis was the only predictor to show clinically important differences. Age was only a significant predictor in the rural population, but again did not show clinically meaningful differences. Studies of children in this age group have demonstrated that HRQOL decreases, not increases, with age [46], which also questions the positive association that was found with age and total HRQOL, psychosocial health, and social functioning. Being a visible minority was not a significant predictor of HRQOL in either population. These findings support other studies assessing HRQOL, where the relationship between intrapersonal variables and the dependent variable have varied [6,7,11,12].

High or medium household income positively predicted five out of the six HRQOL indices in the rural population, all showing a clinically meaningful difference, demonstrating the effects household income can have on HRQOL. However, this pattern was not evident within the urban/suburban population. The finding that household income was a significant predictor of HRQOL only in the rural population demonstrates how certain factors can have a different effect on health outcomes in different environmental settings (i.e., different geographical contexts). 

Two variables that can be considered to be predictors of socio-economic status are parents having post-secondary education and whether or not they are employed. Some results showed negative effects on HRQOL indices when a mother had post-secondary education and was employed, while others showed education as a negative predictor and employment as a positive in fathers. The mix of findings is consistent in both urban/suburban and rural analyses. Some research has shown that children whose mothers stay home have better long term educational outcomes [47]. Although there is not substantial research based evidence supporting better health outcomes for children who have stay at home mothers, a national survey of American parents shows 60% of parents believe children are better off when a parent stays at home [48]. Socio-economic measures, such as parental employment, education, and household income, are important to include, because, as described by Varni and colleagues, near-poor and poor children are approximately three times more likely to have an unmet health care need [30]. Clinically meaningful differences of interpersonal variables from parent surveys may not have been detected due to lower response rates in questions that are associated with socio-economic status, this may also have contributed to the mixed findings throughout.

When analyses were conducted for each of the PedsQL indices, at least one physical environment variable was a significant predictor of every HRQOL outcome in the urban/suburban population. Interestingly, in the rural population natural environment variables were only significant for social functioning. This was potentially due to the little variation in natural environment features that exist in the rural communities. However, it is important to note that none of these significant predictors saw clinically meaningful differences in HRQOL outcomes.

Blue space in general has not been studied extensively in relation to children’s health. Many studies tend to focus on how green spaces or green features of the environment affect a particular outcome. However, it is important to view nature as a more holistic measure, including all forms, not just those that are considered to be “green”. The percentage of water area around a participant’s home was always a negative predictor of HRQOL in the urban/suburban population, however, was both a positive and negative predictor in the rural analysis. The inclusion of all water features within each study region could be the reason behind the negative association. Not all water features within a participant’s home are necessarily “clean” or desirable locations for anyone to frequent. Parental perceptions of water areas being unsafe to play in or near could potentially affect HRQOL scores. Understanding parental perceptions of nature spaces is an important future direction of the current study. The inconsistency in positive and negative relationships in the rural population requires further analysis to provide a meaningful explanation.

The percentage of park space was a positive significant predictor in three out of six HRQOL outcomes in the urban/suburban population, although a clinically meaningful difference was never found. This supports other studies that have found significant relationships between parks and mental health outcomes [14,49,50]. Using a single measure to represent all types of parks, including formally-designed public parks, dense forests, and open fields, could be the reason for the inconclusive findings in the rural population. Future research should consider creating multiple classifications of parks to better understand how the different types of parks impact HRQOL.

Similar to the percentage of park and water space, the urban/suburban population found significant negative relationships with five out of six HRQOL outcomes and at least one NDVI measure. In the rural analysis, however, NDVI was a positive significant predictor for just social functioning. Some studies using NDVI as a measure of green space have also come to similar conclusions of no significance to mental health outcomes [51]. Measures, including proximity to, use of, or time spent in green space have seen more success in finding positive significant relationships [4,12,51,52]. More complex measures of nature have also found positive significant relationships with HRQOL measures [11].

All three measures of the natural environment found some relationship with HRQOL outcomes. However, the coefficients for each of these relationships were almost always less than one, demonstrating a lack of clinically meaningful differences. Therefore, these findings were not strong enough to conclude a meaningful relationship between the accessibility to nature and children’s HRQOL. There is a significant base in the literature to support that differences in geographical context can effect a variety of health outcomes [53,54,55]. In the current study, the level of urbanicity was never a significant predictor of HRQOL. The region of Ontario in the rural analysis also never significantly predicted HRQOL outcomes. These inconclusive findings between geographical context and HRQOL outcomes could be attributed to the built environment of the study region. Truly rural home locations were identified, however, some of the urban areas in the study may not reflect a truly urban area. The major city that was included in this study had significant tree coverage and natural spaces throughout its downtown core, which was considered to be the urban area. However, the differences that do exist between each analysis provide reason to further investigate nature’s effects on these subpopulations. These somewhat inconclusive findings demonstrate the need to use exposure to nature as the next step in assessing the relationship between nature and children’s HRQOL. Exposure is a more accurate representation of a child’s actual interactions with particular spaces, as opposed to assessing the opportunity structure around their home.

### 4.1. Policy and Practice

Although the findings of the current study do not strongly support a definitive relationship one way or the other, there are recommendations that can be made for policy makers and practitioners. The relationships that were found can support the development of programs that focus on getting children outdoors in nature, something that can be achieved through a number of avenues. School boards and public health officials can make it part of their mandate to promote and to develop strategies that get children outdoors, while still accomplishing other primary objectives (e.g., curriculum). Outdoor learning has been shown to positively improve cognitive functioning as well as other measures of health [2]. Simple changes in policy and practice can also help to add other streams of research opportunities in assessing children’s health in relation to nature, through exposure and engagement. The differences that exist in the variables that significantly predicted HRQOL scores in the two populations demonstrates the potential to target certain child populations differently. Policy makers and practitioners should take into consideration where a child lives and the intrapersonal, interpersonal, and physical environment variables that contribute most to their state of well-being.

### 4.2. Strengths, Limitations, and Future Directions

The STEAM protocol provides rich data assessing the healthy behaviours of children in relation to where they live. Methodological strengths include the use of GPS for identifying precise home locations of each child. Exact home locations provide a much more accurate representation of children’s habitual environments than cannot be discerned from commonly-used address proxies such as postal/zip codes, especially in the rural and remote areas of Canada where one postal code can represent hundreds of square kilometres [56]. In addition, multiple data sources were used to compile the most comprehensive park and water layers for natural environmental variables. Although surveys do not allow for individual children’s experiences and opinions to be expressed, they provide large scale data that is important for informing policy and practice [57]. The PedsQL supports the simple collection of information that provide insights into factors influencing a child’s state of well-being. Unlike physical health, mental health is not easily assessed, measured, and defined. Using a tool that can collect information on four key variables that contribute to a child’s overall mental health creates a simple way to investigate the well-being of an individual. Time, financial, ethical, and recruitment constraints do not allow for a measure of mental illness to be used in an elementary school setting. Therefore, the tool that was used in the current study is an effective way to assess a variety of functioning abilities of a child that influence their state of well-being.

It is recommended that using exposure to natural environments to assess the interactions with nature be used in future research. The findings here and in other studies demonstrate that using accessibility to measure nature interactions has its limitations. The inconclusive findings of the current study support that access or opportunity does not necessarily translate into the use of a particular space. Findings from McCracken and colleagues also support this limitation of accessibility, where their results demonstrated that significant relationships were found for time spent in urban green spaces, but not measures of residential green space [12]. Time spent allows for a greater consideration of individual agency of children [17], especially when children potentially have greater limitations in accessing spaces in their neighbourhood environments.

Creators of the PedsQL strongly recommend that whenever possible the parent proxy-report be used in combination with the child self-report [31]. Child self-reports commonly result in more under or over reporting of health functions, supporting the need to include the parent-proxy report. The current study did not provide the parent proxy in the parent survey, as the STEAM project is an interdisciplinary study where HRQOL was a secondary purpose. Research funding was not explicitly available for a study of HRQOL. Future research should consider the utilization of both tools to confirm the HRQOL scores. More time between data collection is needed in order to detect true differences in HRQOL. The current protocol did measure HRQOL at two different times (a maximum of six months apart), however, no significant changes were observed between the two data collection periods, suggesting that more time is needed to see the observable differences. Measuring the effects of nature on HRQOL is a passive intervention, which also supports the need for greater time between baseline and follow-up to detect changes in the dependent variables. The socio-ecological model describes a variety of variables that can affect a child’s state of well-being. However, the current study was limited in its lack of information regarding participant’s behaviours, attitudes, and limitations surrounding their HRQOL. For example, another factor that may potentially influence HRQOL is the quality of the school, as it has been shown that lower school quality can result in poor health outcomes in children [58]. Although likely being impossible to assess every factor that affects a child’s state of well-being, future research should include more independent variables that are influencing HRQOL found within a socio-ecological model.

## 5. Conclusions

This study has identified the influence that different types of nature have on children’s HRQOL. The patterns between significant independent variables are noticeably different between the urban/suburban and rural populations. These differences need to be further investigated while using actual exposure to nature in different geographical contexts. Furthermore, the lack of strong relationships between the physical environment variables and HRQOL outcomes also demonstrates a need to measure the actual exposure. Future research should move towards assessing children’s health and nature through exposure and engagement to more accurately represent the dose and the duration of nature that is required to see significant effects on HRQOL.

## Figures and Tables

**Table 1:** Minimal clinically important difference for health-related quality of life (HRQOL) indices.

HRQOL Indices	Minimal Clinically Important Difference
Total Scale Score	4.57
Psychosocial Health	5.29
Physical Functioning	3.89
Emotional Functioning	5.83
Social Functioning	5.46
School Functioning	4.79

**Table 2:** Descriptive statistics of sample.

	All Participants		Urban/Suburban		Rural	
	*n*	Mean (SD) or % of *n*	*n*	Mean (SD) or % of *n*	*n*	Mean (SD) or % of *n*
**Intrapersonal**						
Boys	379	44.5	195	41.8	184	47.9
Girls	472	55.5	272	58.2	200	52.1
*Age*	851	11.1 (0.984)	467	11.3 (0.904)	11.0	(1.0)
8	6	0.7	-	-	6	1.6
9	37	4.3	10	2.1	27	7.0
10	148	17.4	76	16.3	72	18.8
11	367	43.1	205	43.9	162	42.2
12	236	27.7	138	29.6	98	25.5
13	54	6.3	37	7.9	17	4.4
14	3	0.4	1	0.2	2	0.5
Visible Minority	234	27.5	154	33.0	80	20.8
**Interpersonal**						
Lone Parent Household	260	30.6	112	24.0	148	38.5
Live in more than 1 home	135	15.9	69	14.8	66	17.2
No siblings	117	13.7	67	14.3	50	13.0
Mother Post Secondary	524	61.6	271	58.0	253	65.9
Father Post Secondary	445	52.3	248	53.1	197	51.3
Mother Employed	524	61.6	243	52.0	281	73.2
Father Employed	578	67.9	284	60.8	294	76.6
Household Income	480	-	245	-	235	-
Low: <$70,000	159	18.7	86	18.4	73	19.0
Medium: 70,000 to $119,999	164	19.3	86	18.4	78	20.3
High: ≥ $120,000	157	18.4	73	15.6	84	21.9
**Physical Environment**						
Park	851	7.4 (8.6)	467	11.4 (9.1)	384	2.5 (4.4)
Water	851	1.5 (5.3)	467	1.0 (2.6)	384	2.0 (7.4)
Grass & Shrubbery	851	56.2 (20.1)	467	67.8 (12.1)	384	42.1 (18.8)
Dense Vegetation	851	38.4 (21.7)	467	26.2 (12.3)	384	53.3 (21.3)
Urban	79	9.3	79	16.9	-	-
Suburban	388	45.6	388	83.1	-	-
Rural	384	45.1	-	-	384	100
Northwestern Ontario	129	15.2	-	-	129	100
Southwestern Ontario	722	84.8	467	64.7	255	35.3
**Health-related Quality of Life**						
Total Scale Score	850	79.7 (13.2)	466	80.1 (13.6)	384	79.2 (12.8)
Psychosocial Health (/100)	851	76.3 (15.1)	467	76.9 (15.5)	384	75.6 (14.5)
Physical Functioning (/100)	850	85.9 (13.9)	466	86.0 (13.9)	384	85.8 (13.9)
Emotional Functioning (/100)	847	73.0 (20.0)	464	73.0 (20.5)	383	73.0 (19.5)
Social Functioning (/100)	847	81.7 (17.7)	465	82.9 (17.2)	382	80.2 (18.3)
School Functioning (/100)	850	74.1 (16.9)	467	74.7 (15.5)	383	73.5 (16.0)

**Table 3:** Results of full models assessing associations between all independent variables and HRQOL indices at a 500M buffer in the urban/suburban population.

Variable	Total Scale Score		Psychosocial Health		Physical Functioning		Emotional Functioning		Social Functioning		School Functioning	
	β (SE)	*p*	β (SE)	*p*	β (SE)	*p*	β (SE)	*p*	β (SE)	*p*	β (SE)	*p*
**Intrapersonal**												
Boy (ref: girl)	−0.58 (1.28)	0.649	−0.15 (1.47)	0.916	−1.56 (1.31)	0.236	3.42 (1.95)	0.080	−1.69 (1.63)	0.300	−2.03 (1.66)	0.220
Age (years)	0.10 (0.71)	0.878	−0.07 (0.81)	0.932	0.34 (0.72)	0.634	−0.16 (1.08)	0.880	−0.29 (0.90)	0.743	0.37 (0.91)	0.687
Visible Minority (ref: no)	0.48 (1.43)	0.736	1.68 (1.64)	0.306	−1.83 (1.46)	0.212	0.80 (2.17)	0.713	2.19 (1.81)	0.229	2.10 (1.85)	0.257
**Interpersonal**												
Lone Parent Household (ref: no)	0.64 (1.74)	0.713	1.08 (2.00)	0.589	−0.12 (1.79)	0.944	3.63 (2.65)	0.171	1.37 (2.21)	0.534	−1.86 (2.26)	0.412
Live in more than 1 home (ref: yes)	3.21 (1.99)	0.107	3.93 (2.29)	0.087	1.65 (2.04)	0.418	4.39 (3.03)	0.147	1.82 (2.53)	0.471	5.74 (2.58)	**0.027**
Siblings (ref: no)	1.95 (1.83)	0.287	2.62 (2.10)	0.213	0.63 (1.88)	0.735	4.41 (2.80)	0.116	5.28 (2.34)	**0.025**	−1.67 (2.37)	0.481
Mother Post-secondary (ref: no)	−1.71 (2.04)	0.402	−1.32 (2.34)	0.571	−2.06 (2.09)	0.325	−2.15 (3.10)	0.488	0.44 (2.59)	0.865	−2.40 (2.64)	0.363
Father Post-secondary (ref: no)	1.60 (1.92)	0.406	1.79 (2.20)	0.418	1.50 (1.98)	0.447	1.17 (2.93)	0.688	4.82 (2.45)	0.050	−1.02 (2.49)	0.682
Mother Employed (ref: no)	−1.40 (1.82)	0.440	−1.31 (2.09)	0.530	−1.49 (1.87)	0.424	−3.11 (2.76)	0.262	−0.14 (2.31)	0.951	−0.76 (2.36)	0.745
Father Employed (ref: no)	−0.83 (2.86)	0.771	−0.85 (3.29)	0.795	−1.03 (2.94)	0.726	−1.57 (4.35)	0.718	−3.80 (3.63)	0.297	3.18 (3.71)	0.391
Household Income (ref: low)												
Medium	−0.28 (2.25)	0.899	−1.32 (2.58)	0.609	1.94 (2.31)	0.400	−1.40 (3.42)	0.682	2.07 (2.86)	0.468	−5.01 (2.91)	0.086
High	0.85 (2.56)	0.739	0.06 (2.94)	0.982	2.52 (2.62)	0.337	−2.28 (3.90)	0.558	2.51 (3.26)	0.441	−0.42 (3.31)	0.897
**Physical Environment**												
Park	0.18 (0.09)	**0.039**	0.19 (0.10)	0.062	0.18 (0.09)	**0.047**	0.24 (0.13)	0.084	0.27 (0.11)	**0.018**	0.06 (0.11)	0.593
Water	−1.13 (0.29)	**0.000**	−1.09 (0.34)	**0.001**	−1.21 (0.30)	**0.000**	−1.26 (0.45)	**0.005**	−1.43 (0.37)	**0.000**	−0.57 (0.38)	0.135
Grass & Shrubbery	−0.28 (0.11)	**0.012**	−0.27 (0.12)	**0.033**	−0.32 (0.11)	**0.006**	−0.11 (0.17)	0.521	−0.31 (0.14)	**0.028**	−0.40 (0.14)	**0.006**
Dense Vegetation	−0.15 (0.11)	0.170	−0.12 (0.13)	0.325	−0.22 (0.11)	0.052	0.02 (0.17)	0.876	−0.21 (0.14)	0.144	−0.18 (0.14)	0.202
Urbanicity (ref: urban)	2.00 (1.72)	0.246	2.19 (1.98)	0.269	1.58 (1.77)	0.372	1.11 (2.62)	0.671	1.18 (2.19)	0.590	4.28 (2.23)	0.056
Constant	98.31 (13.81)	**0.000**	93.46 (15.82)	**0.000**	109.70 (14.18)	**0.000**	76.57 (21.05)	**0.000**	103.10 (17.6)	**0.000**	99.03 (17.84)	**0.000**
R^2^	0.0855	**0.026**	0.0748	0.085	0.0854	**0.027**	0.0720	0.117	0.0829	**0.037**	0.0782	0.060

All bold numbers represent significant **β** values at the *p* < 0.05 or *p* <0.01.

**Table 4:** Results of full models assessing associations between all independent variables and HRQOL indices at a 500M buffer in the rural population.

Variable	Total Scale Score		Psychosocial Health		Physical Functioning		Emotional Functioning		Social Functioning		School Functioning	
	β (SE)	*p*	β (SE)	*p*	β (SE)	*p*	β (SE)	*p*	β (SE)	*p*	β (SE)	*p*
**Intrapersonal**												
Boy (ref: girl)	1.37 (1.37)	0.317	1.30 (1.54)	0.399	1.39 (1.49)	0.353	6.77 (2.07)	**0.001**	−0.31 (1.91)	0.870	−2.49 (1.71)	0.145
Age (years)	1.28 (0.65)	0.050	1.59 (0.73)	**0.031**	0.66 (0.71)	0.353	0.97 (0.98)	0.325	3.09 (0.90)	**0.001**	0.77 (0.81)	0.344
Visible Minority (ref: no)	0.23 (1.84)	0.900	0.29 (2.08)	0.887	0.19 (2.01)	0.921	−2.93 (2.78)	0.293	0.30 (2.56)	0.906	3.51 (2.30)	0.128
**Interpersonal**												
Lone Parent Household (ref: no)	−2.49 (1.72)	0.150	−1.87 (1.94)	0.335	−3.68 (1.87)	0.051	−2.97 (2.59)	0.253	−1.19 (2.39)	0.618	−1.48 (2.15)	0.491
Live in more than 1 home (ref: yes)	−0.62 (1.88)	0.741	1.44 (2.12)	0.498	−4.48 (2.05)	**0.030**	1.80 (2.84)	0.525	3.17 (2.62)	0.228	−0.34 (2.36)	0.885
Siblings (ref: no)	−1.00 (2.01)	0.618	−1.59 (2.26)	0.482	0.09 (2.19)	0.967	−3.59 (3.02)	0.236	−1.65 (2.79)	0.555	0.50 (2.50)	0.842
Mother Post-secondary (ref: no)	0.21 (1.86)	0.907	1.15 (2.10)	0.583	−1.64 (2.03)	0.421	0.65 (2.81)	0.817	1.23 (2.62)	0.639	1.47 (2.33)	0.529
Father Post-secondary (ref: no)	−0.39 (1.60)	0.805	−1.04 (1.80)	0.565	0.75 (1.74)	0.667	−3.08 (2.41)	0.203	−1.00 (2.23)	0.652	0.94 (2.00)	0.639
Mother Employed (ref: no)	−0.22 (2.20)	0.920	0.29 (2.48)	0.906	−1.15 (2.40)	0.631	0.80 (3.31)	0.809	1.02 (3.06)	0.738	−0.35 (2.77)	0.899
Father Employed (ref: no)	4.67 (2.80)	0.097	4.72 (3.15)	0.135	4.82 (3.05)	0.116	5.75 (4.22)	0.174	4.22 (3.89)	0.279	5.06 (3.56)	0.156
Household Income (ref: low)												
Medium	3.45 (2.24)	0.124	4.92 (2.52)	0.052	0.72 (2.44)	0.767	6.78 (3.37)	**0.045**	6.90 (3.11)	**0.027**	1.21 (2.79)	0.665
High	5.87 (2.29)	**0.011**	7.25 (2.58)	**0.005**	3.24 (2.50)	0.195	4.87 (3.46)	0.161	11.11 (3.20)	**0.001**	5.96 (2.86)	**0.038**
**Physical Environment**												
Park	0.00 (0.16)	0.984	0.01 (0.18)	0.948	−0.03 (0.18)	0.853	0.11 (0.25)	0.640	−0.20 (0.23)	0.377	0.08 (0.21)	0.695
Water	0.13 (0.14)	0.345	0.13 (0.16)	0.418	0.14 (0.15)	0.351	−0.01 (0.21)	0.961	0.29 (0.20)	0.145	0.12 (0.17)	0.480
Grass & Shrubbery	0.20 (0.16)	0.228	0.22 (0.19)	0.235	0.16 (0.18)	0.378	−0.10 (0.25)	0.674	0.48 (0.23)	**0.040**	0.32 (0.21)	0.126
Dense Vegetation	0.19 (0.14)	0.191	0.19 (0.16)	0.246	0.19 (0.16)	0.227	−0.12 (0.22)	0.573	0.41 (0.20)	**0.047**	0.31 (0.18)	0.089
Region of Ontario (ref: south)	0.16 (1.88)	0.929	0.02 (2.12)	0.992	0.41 (2.05)	0.839	1.47 (2.84)	0.605	−1.85 (2.62)	0.482	0.49 (2.35)	0.833
Constant	39.92 (17.17)	**0.021**	28.83 (19.34)	0.137	61.07 (18.72)	**0.001**	64.05 (25.86)	**0.014**	−8.13 (23.88)	0.733	26.26 (21.55)	0.224
R^2^	0.0666	0.380	0.0761	0.210	0.0599	0.528	0.0859	0.101	0.1177	**0.004**	0.0761	0.213

All bold numbers represent significant **β** values at the *p* < 0.05 or *p* < 0.01.

## Supplementary Materials

The following are available online at http://www.mdpi.com/1660-4601/15/6/1072/s1, Table S1: Results of full models assessing associations between all independent variables and HRQOL indices at an 800M buffer in the urban/suburban population, Table S2: Results of full models assessing associations between all independent variables and HRQOL indices at an 800M buffer in the rural population, Table S3: Results of full models assessing associations between all independent variables and HRQOL indices at a 1000M buffer in the urban/suburban population, Table S4: Results of full models assessing associations between all independent variables and HRQOL indices at a 1000M buffer in the rural population, Table S5: Results of full models assessing associations between all independent variables and HRQOL indices at a 1600M buffer in the urban/suburban population, Table S6: Results of full models assessing associations between all independent variables and HRQOL indices at a 1600M buffer in the rural population.
