# Supplementary material for: Children and Nature: Linking Accessibility of Natural Environments and Children’s Health-Related Quality of Life
Source: Int J Environ Res Public Health. 2018 May 25;15(6):1072. doi: 10.3390/ijerph15061072 (PMC6025036; doi:10.3390/ijerph15061072)
Supplement: Supplementary file 1 [file ijerph-15-01072-s001.pdf]

Supplementary Table 1: Results of full models assessing associations between all independent variables and HRQOL indices at an 800m buffer in the urban/suburban population

Variable	Total Scale Score		Psychosocial Health		Physical Functioning		Emotional Functioning		Social Functioning		School Functioning	
	β (SE)	*p*	β (SE)	*p*	β (SE)	*p*	β (SE)	*p*	β (SE)	*p*	β (SE)	*p*
**Intrapersonal**												
Boy (ref: girl)	-0.65 (1.29)	0.616	-0.22 (1.48)	0.880	-1.64 (1.32)	0.218	3.45 (1.96)	0.080	-1.77 (1.64)	0.283	-2.18 (1.66)	0.192
Age (years)	0.00 (0.72)	0.989	-0.14 (0.82)	0.858	0.20 (0.74)	0.788	-0.25 (1.10)	0.818	-0.45 (0.92)	0.625	0.37 (0.93)	0.687
Visible Minority (ref: no)	0.87 (1.45)	0.547	2.05 (1.66)	0.216	-1.39 (1.48)	0.349	1.20 (2.19)	0.584	2.75 (1.84)	0.136	2.25 (1.86)	0.229
**Interpersonal**												
Lone Parent Household (ref: no)	0.50 (1.76)	0.773	0.89 (2.02)	0.660	-0.11 (1.81)	0.948	3.13 (2.67)	0.242	1.18 (2.24)	0.598	-1.75 (2.27)	0.440
Live in more than 1 home (ref: yes)	2.99 (2.01)	0.138	3.69 (2.31)	0.110	1.48 (2.06)	0.471	3.91 (3.04)	0.200	1.49 (2.55)	0.560	5.82 (2.59)	**0.025**
Siblings (ref: no)	1.96 (1.85)	0.290	2.61 (2.12)	0.221	0.70 (1.9)	0.711	4.51 (2.82)	0.111	5.35 (2.37)	**0.025**	-1.89 (2.39)	0.428
Mother Post-secondary (ref: no)	-1.48 (2.06)	0.472	-1.09 (2.36)	0.643	-1.86 (2.11)	0.379	-1.94 (3.12)	0.533	0.78 (2.62)	0.765	-2.24 (2.65)	0.398
Father Post-secondary (ref: no)	1.77 (1.93)	0.360	1.93 (2.21)	0.383	1.70 (1.98)	0.392	1.55 (2.93)	0.595	5.13 (2.46)	**0.037**	-1.26 (2.48)	0.613
Mother Employed (ref: no)	-1.87 (1.84)	0.310	-1.82 (2.11)	0.390	-1.89 (1.89)	0.318	-3.62 (2.79)	0.195	-0.78 (2.34)	0.740	-1.13 (2.38)	0.634
Father Employed (ref: no)	-0.34 (2.89)	0.905	-0.43 (3.32)	0.897	-0.42 (2.97)	0.887	-1.11 (4.37)	0.798	-3.12 (3.67)	0.397	3.29 (3.73)	0.377
Household Income (ref: low)												
Medium	-0.05 (2.27)	0.981	-1.01 (2.60)	0.696	2.04 (2.33)	0.382	-0.75 (3.44)	0.826	2.34 (2.89)	0.418	-4.99 (2.92)	0.089
High	1.03 (2.59)	0.689	0.39 (2.97)	0.894	2.43 (2.66)	0.362	-1.57 (3.93)	0.690	2.97 (3.30)	0.368	-0.60 (3.34)	0.856
**Physical Environment**												
Park	0.11 (0.09)	0.196	0.11 (0.10)	0.289	0.14 (0.09)	0.123	0.10 (0.14)	0.455	0.21 (0.11)	0.072	0.01 (0.11)	0.905
Water	-0.92 (0.38)	**0.015**	-0.86 (0.43)	**0.047**	-1.03 (0.39)	**0.008**	-0.96 (0.57)	0.096	-1.02 (0.48)	**0.034**	-0.63 (0.49)	0.195
Grass & Shrubbery	-0.29 (0.14)	**0.044**	-0.28 (0.16)	0.081	-0.30 (0.14)	**0.04**	-0.16 (0.21)	0.446	-0.30 (0.18)	0.091	-0.40 (0.18)	**0.031**
Dense Vegetation	-0.18 (0.14)	0.193	-0.16 (0.16)	0.3	-0.22 (0.14)	0.122	-0.04 (0.21)	0.841	-0.24 (0.17)	0.165	-0.20 (0.18)	0.249
Urbanicity (ref: urban)	1.78 (1.77)	0.316	1.95 (2.04)	0.340	1.42 (1.82)	0.434	0.89 (2.69)	0.739	1.24 (2.26)	0.584	3.67 (2.29)	0.110
Constant	100.18 (16.14)	**0.000**	96.13 (18.49)	**0.000**	109.4 (16.56)	**0.000**	83.59 (24.46)	**0.001**	104.25 (20.54)	**0.000**	100.12 (20.77)	**0.000**
R^2	0.062	0.254	0.056	0.402	0.064	0.229	0.057	0.374	0.060	0.301	0.067	0.175

Supplementary Table 2: Results of full models assessing associations between all independent variables and HRQOL indices at an 800m buffer in the rural population

Variable	Total Scale Score		Psychosocial Health		Physical Functioning		Emotional Functioning		Social Functioning		School Functioning	
	β (SE)	*p*	β (SE)	*p*	β (SE)	*p*	β (SE)	*p*	β (SE)	*p*	β (SE)	*p*
**Intrapersonal**												
Boy (ref: girl)	1.21 (1.35)	0.373	1.17 (1.53)	0.443	1.15 (1.48)	0.437	6.72 (2.05)	**0.001**	-0.67 (1.90)	0.725	-2.47 (1.68)	0.144
Age (years)	1.29 (0.64)	**0.046**	1.61 (0.73)	**0.028**	0.65 (0.70)	0.358	0.94 (0.98)	0.334	3.14 (0.90)	**0.001**	0.82 (0.80)	0.308
Visible Minority (ref: no)	0.25 (1.83)	0.888	0.31 (2.06)	0.881	0.24 (1.99)	0.901	-3.03 (2.76)	0.274	0.46 (2.56)	0.856	3.51 (2.27)	0.123
**Interpersonal**												
Lone Parent Household (ref: no)	-2.60 (1.71)	0.131	-1.97 (1.93)	0.308	-3.80 (1.86)	**0.042**	-3.01 (2.59)	0.246	-1.44 (2.39)	0.548	-1.48 (2.13)	0.488
Live in more than 1 home (ref: yes)	-0.54 (1.87)	0.771	1.47 (2.11)	0.486	-4.32 (2.04)	**0.035**	1.72 (2.83)	0.543	3.10 (2.62)	0.238	-0.11 (2.33)	0.960
Siblings (ref: no)	-1.01 (2.01)	0.615	-1.61 (2.27)	0.478	0.09 (2.19)	0.964	-3.97 (3.03)	0.191	-1.64 (2.81)	0.561	0.84 (2.49)	0.737
Mother Post-secondary (ref: no)	0.40 (1.86)	0.829	1.30 (2.10)	0.534	-1.39 (2.02)	0.491	0.81 (2.81)	0.773	1.31 (2.63)	0.617	1.68 (2.31)	0.466
Father Post-secondary (ref: no)	-0.30 (1.58)	0.850	-0.90 (1.79)	0.616	0.76 (1.72)	0.657	-2.92 (2.39)	0.224	-0.78 (2.22)	0.723	0.93 (1.97)	0.635
Mother Employed (ref: no)	0.13 (2.20)	0.950	0.63 (2.48)	0.798	-0.75 (2.39)	0.752	1.04 (3.32)	0.753	1.44 (3.07)	0.638	-0.07 (2.74)	0.979
Father Employed (ref: no)	4.60 (2.76)	0.097	4.77 (3.12)	0.127	4.54 (3.01)	0.133	5.55 (4.18)	0.185	4.28 (3.87)	0.269	5.31 (3.50)	0.130
Household Income (ref: low)												
Medium	3.55 (2.22)	0.112	4.97 (2.51)	**0.048**	0.90 (2.42)	0.709	6.81 (3.36)	**0.043**	7.00 (3.11)	**0.025**	1.22 (2.76)	0.658
High	5.67 (2.27)	**0.013**	7.01 (2.57)	**0.007**	3.12 (2.48)	0.208	4.80 (3.44)	0.165	10.77 (3.20)	**0.001**	5.60 (2.82)	**0.048**
**Physical Environment**												
Park	0.31 (0.24)	0.191	0.29 (0.27)	0.286	0.36 (0.26)	0.163	0.27 (0.36)	0.452	0.22 (0.34)	0.503	0.28 (0.30)	0.346
Water	0.22 (0.16)	0.156	0.21 (0.18)	0.226	0.24 (0.17)	0.169	-0.08 (0.24)	0.715	0.35 (0.22)	0.111	0.41 (0.19)	**0.039**
Grass & Shrubbery	0.26 (0.19)	0.189	0.26 (0.22)	0.243	0.25 (0.02)	0.239	-0.28 (0.30)	0.344	0.44 (0.27)	0.108	0.66 (0.24)	**0.008**
Dense Vegetation	0.27 (0.17)	0.112	0.24 (0.19)	0.199	0.31 (0.18)	0.092	-0.26 (0.25)	0.311	0.40 (0.23)	0.093	0.62 (0.21)	**0.003**
Region of Ontario (ref: south)	0.79 (1.92)	0.682	0.58 (2.17)	0.789	1.15 (2.09)	0.583	1.38 (2.90)	0.634	-1.01 (2.69)	0.707	1.36 (2.39)	0.569
Constant	31.83 (19.53)	0.104	23.00 (22.04)	0.297	49.27 (21.26)	**0.021**	78.54 (29.49)	**0.008**	-7.91 (27.32)	0.772	-6.71 (24.37)	0.783
R^2	0.075	0.227	0.080	0.149	0.070	0.310	0.089	0.076	0.115	**0.005**	0.095	**0.044**

Supplementary Table 3: Results of full models assessing associations between all independent variables and HRQOL indices at a 1000m buffer in the urban/suburban population

Variable	Total Scale Score		Psychosocial Health		Physical Functioning		Emotional Functioning		Social Functioning		School Functioning	
	β (SE)	*p*	β (SE)	*p*	β (SE)	*p*	β (SE)	*p*	β (SE)	*p*	β (SE)	*p*
**Intrapersonal**												
Boy (ref: girl)	-0.76 (1.30)	0.555	-0.34 (1.48)	0.816	-1.76 (1.33)	0.188	3.38 (1.97)	0.087	-1.93 (1.65)	0.240	-2.31 (1.67)	0.167
Age (years)	0.05 (0.72)	0.938	-0.10 (0.83)	0.900	0.24 (0.74)	0.744	-0.17 (1.10)	0.877	-0.45 (0.92)	0.624	0.44 (0.93)	0.635
Visible Minority (ref: no)	0.90 (1.45)	0.534	2.07 (1.66)	0.214	-1.34 (1.49)	0.371	1.20 (2.20)	0.586	2.84 (1.84)	0.123	2.21 (1.87)	0.238
**Interpersonal**												
Lone Parent Household (ref: no)	0.59 (1.77)	0.737	0.91 (2.03)	0.654	0.10 (1.82)	0.956	3.10 (2.68)	0.247	1.34 (2.24)	0.550	-1.84 (2.28)	0.419
Live in more than 1 home (ref: yes)	3.16 (2.01)	0.117	3.81 (2.30)	0.099	1.73 (2.06)	0.402	4.02 (3.04)	0.186	1.67 (2.55)	0.512	5.89 (2.59)	**0.023**
Siblings (ref: no)	1.82 (1.86)	0.328	2.46 (2.13)	0.249	0.57 (1.91)	0.762	4.29 (2.83)	0.130	5.26 (2.37)	**0.027**	-2.03 (2.39)	0.395
Mother Post-secondary (ref: no)	-1.23 (2.06)	0.552	-0.80 (2.36)	0.734	-1.66 (2.12)	0.434	-1.59 (3.12)	0.611	1.02 (2.62)	0.695	-1.99 (2.65)	0.454
Father Post-secondary (ref: no)	1.83 (1.94)	0.346	1.98 (2.21)	0.370	1.78 (1.99)	0.371	1.62 (2.93)	0.579	5.20 (2.46)	**0.035**	-1.26 (2.49)	0.613
Mother Employed (ref: no)	-2.17 (1.84)	0.239	-2.16 (2.11)	0.306	-2.10 (1.89)	0.268	-4.00 (2.78)	0.152	-1.06 (2.33)	0.649	-1.51 (2.37)	0.525
Father Employed (ref: no)	-0.26 (2.89)	0.927	-0.41 (3.32)	0.900	-0.23 (2.97)	0.938	-1.14 (4.37)	0.794	-3.06 (3.67)	0.405	3.30 (3.73)	0.376
Household Income (ref: low)												
Medium	-0.11 (2.29)	0.961	-1.10 (2.62)	0.675	2.05 (2.35)	0.384	-0.76 (3.46)	0.824	2.35 (2.90)	0.418	-5.27 (2.94)	0.074
High	1.08 (2.59)	0.677	0.57 (2.97)	0.847	2.22 (2.66)	0.405	-1.24 (3.93)	0.751	2.98 (3.29)	0.366	-0.40 (3.33)	0.903
**Physical Environment**												
Park	0.13 (0.09)	0.174	0.12 (0.11)	0.245	0.15 (0.09)	0.128	0.1 (0.14)	0.480	0.24 (0.12)	**0.043**	0.02 (0.12)	0.865
Water	-0.82 (0.40)	**0.040**	-0.77 (0.46)	0.091	-0.90 (0.41)	**0.028**	-0.83 (0.60)	0.170	-1.00 (0.50)	**0.049**	-0.51 (0.51)	0.324
Grass & Shrubbery	-0.28 (0.15)	0.07	-0.30 (0.17)	0.082	-0.23 (0.15)	0.137	-0.17 (0.23)	0.467	-0.32 (0.19)	0.1	-0.44 (0.19)	**0.025**
Dense Vegetation	-0.20 (0.14)	0.154	-0.22 (0.16)	0.176	-0.18 (0.14)	0.22	-0.10 (0.21)	0.635	-0.28 (0.18)	0.12	-0.28 (0.18)	0.127
Urbanicity (ref: urban)	1.87 (1.82)	0.305	2.10 (2.08)	0.313	1.37 (1.86)	0.461	0.99 (2.75)	0.717	1.34 (2.30)	0.560	3.94 (2.34)	0.093
Constant	99.11 (16.73)	**0.000**	97.94 (19.16)	**0.000**	102.79 (17.18)	**0.000**	84.42 (25.31)	**0.001**	105.26 (21.21)	**0.000**	103.93 (21.51)	**0.000**
R^2	0.057	0.378	0.052	0.497	0.056	0.389	0.054	0.462	0.060	0.309	0.064	0.224

Supplementary Table 4: Results of full models assessing associations between all independent variables and HRQOL indices at a 1000m buffer in the rural population

Variable	Total Scale Score		Psychosocial Health		Physical Functioning		Emotional Functioning		Social Functioning		School Functioning	
	β (SE)	*p*	β (SE)	*p*	β (SE)	*p*	β (SE)	*p*	β (SE)	*p*	β (SE)	*p*
**Intrapersonal**												
Boy (ref: girl)	1.21 (1.35)	0.368	1.19 (1.52)	0.434	1.13 (1.47)	0.440	6.94 (2.04)	**0.001**	-0.69 (1.89)	0.715	-2.62 (1.67)	0.117
Age (years)	1.32 (0.64)	**0.042**	1.64 (0.73)	**0.025**	0.66 (0.70)	0.344	0.94 (0.98)	0.338	3.17 (0.90)	**0.001**	0.86 (0.80)	0.280
Visible Minority (ref: no)	0.22 (1.82)	0.901	0.28 (2.06)	0.889	0.20 (1.99)	0.919	-3.12 (2.76)	0.260	0.46 (2.55)	0.856	3.54 (2.26)	0.118
**Interpersonal**												
Lone Parent Household (ref: no)	-2.66 (1.71)	0.120	-2.03 (1.93)	0.293	-3.89 (1.86)	**0.038**	-2.87 (2.59)	0.268	-1.55 (2.39)	0.517	-1.70 (2.12)	0.423
Live in more than 1 home (ref: yes)	-0.47 (1.87)	0.799	1.52 (2.11)	0.470	-4.22 (2.03)	**0.039**	1.79 (2.83)	0.526	3.08 (2.62)	0.240	-0.05 (2.32)	0.980
Siblings (ref: no)	-0.83 (2.00)	0.676	-1.44 (2.26)	0.523	0.28 (2.18)	0.895	-3.70 (3.03)	0.224	-1.52 (2.81)	0.588	0.92 (2.48)	0.708
Mother Post-secondary (ref: no)	0.43 (1.85)	0.815	1.32 (2.09)	0.529	-1.32 (2.02)	0.512	0.88 (2.81)	0.754	1.25 (2.63)	0.634	1.70 (2.30)	0.460
Father Post-secondary (ref: no)	-0.32 (1.57)	0.838	-0.91 (1.78)	0.607	0.73 (1.72)	0.669	-3.16 (2.39)	0.187	-0.72 (2.21)	0.743	1.09 (1.95)	0.575
Mother Employed (ref: no)	-0.03 (2.19)	0.987	0.47 (2.47)	0.849	-0.95 (2.39)	0.690	0.87 (3.32)	0.792	1.21 (3.07)	0.694	-0.18 (2.73)	0.947
Father Employed (ref: no)	5.03 (2.75)	0.069	5.17 (3.11)	0.097	5.01 (3.00)	0.096	5.57 (4.17)	0.182	4.77 (3.85)	0.217	5.92 (3.47)	0.089
Household Income (ref: low)												
Medium	3.71 (2.22)	0.096	5.14 (2.51)	**0.041**	1.07 (2.42)	0.658	6.96 (3.36)	**0.039**	7.20 (3.11)	**0.021**	1.35 (2.75)	0.624
High	5.66 (2.27)	**0.013**	7.00 (2.56)	**0.007**	3.12 (2.47)	0.207	4.95 (3.44)	0.152	10.82 (3.19)	**0.001**	5.37 (2.81)	0.057
**Physical Environment**												
Park	0.29 (0.28)	0.307	0.24 (0.32)	0.443	0.38 (0.31)	0.222	0.13 (0.43)	0.761	0.13 (0.40)	0.734	0.38 (0.36)	0.292
Water	0.32 (0.16)	**0.043**	0.32 (0.18)	0.077	0.33 (0.17)	0.059	0.04 (0.24)	0.844	0.41 (0.22)	0.070	0.52 (0.20)	**0.009**
Grass & Shrubbery	0.40 (0.20)	**0.047**	0.40 (0.23)	0.08	0.40 (0.22)	0.068	-0.09 (0.31)	0.768	0.53 (0.28)	0.064	0.80 (0.25)	**0.002**
Dense Vegetation	0.38 (0.17)	**0.028**	0.35 (0.19)	0.071	0.43 (0.18)	**0.022**	-0.11 (0.26)	0.663	0.44 (0.24)	0.065	0.74 (0.21)	**0.001**
Region of Ontario (ref: south)	0.92 (1.94)	0.635	0.67 (2.20)	0.759	1.36 (2.12)	0.520	1.03 (2.95)	0.727	-0.94 (2.73)	0.729	1.92 (2.41)	0.425
Constant	19.22 (19.90)	0.335	10.84 (22.46)	0.630	35.67 (21.68)	0.101	62.85 (30.12)	**0.038**	-14.09 (27.87)	0.614	-20.2 (24.70)	0.414
R^2	0.078	0.173	0.084	0.115	0.072	0.262	0.088	0.084	0.116	**0.005**	0.103	**0.020**

Supplementary Table 5: Results of full models assessing associations between all independent variables and HRQOL indices at a 1600m buffer in the urban/suburban population

Variable	Total Scale Score		Psychosocial Health		Physical Functioning		Emotional Functioning		Social Functioning		School Functioning	
	β (SE)	*p*	β (SE)	*p*	β (SE)	*p*	β (SE)	*p*	β (SE)	*p*	β (SE)	*p*
**Intrapersonal**												
Boy (ref: girl)	-0.65 (1.30)	0.616	-0.24 (1.49)	0.869	-1.64 (1.34)	0.222	3.48 (1.97)	0.079	-1.82 (1.65)	0.271	-2.23 (1.67)	0.184
Age (years)	0.19 (0.73)	0.793	0.02 (0.83)	0.981	0.38 (0.75)	0.606	-0.09 (1.11)	0.929	-0.38 (0.93)	0.678	0.70 (0.93)	0.456
Visible Minority (ref: no)	0.72 (1.45)	0.621	1.89 (1.66)	0.256	-1.55 (1.49)	0.302	1.08 (2.20)	0.623	2.62 (1.84)	0.155	2.03 (1.87)	0.278
**Interpersonal**												
Lone Parent Household (ref: no)	0.40 (1.78)	0.820	0.71 (2.04)	0.726	-0.09 (1.83)	0.961	3.06 (2.69)	0.255	1.06 (2.25)	0.637	-2.13 (2.29)	0.354
Live in more than 1 home (ref: yes)	3.30 (2.02)	0.104	3.95 (2.31)	0.089	1.87 (2.08)	0.368	4.06 (3.05)	0.184	1.81 (2.55)	0.479	6.14 (2.60)	**0.019**
Siblings (ref: no)	1.78 (1.87)	0.340	2.46 (2.14)	0.251	0.49 (1.92)	0.798	4.24 (2.84)	0.136	5.32 (2.38)	**0.026**	-2.07 (2.40)	0.388
Mother Post-secondary (ref: no)	-1.07 (2.07)	0.604	-0.68 (2.37)	0.773	-1.45 (2.13)	0.496	-1.46 (3.12)	0.638	1.15 (2.61)	0.658	-1.86 (2.65)	0.483
Father Post-secondary (ref: no)	2.14 (1.95)	0.273	2.32 (2.22)	0.298	2.08 (2.00)	0.300	1.84 (2.94)	0.530	5.65 (2.46)	**0.022**	-0.93 (2.50)	0.710
Mother Employed (ref: no)	-2.36 (1.84)	0.201	-2.37 (2.11)	0.261	-2.24 (1.89)	0.236	-4.09 (2.77)	0.142	-1.29 (2.33)	0.579	-1.85 (2.37)	0.435
Father Employed (ref: no)	0.14 (2.90)	0.959	0.01 (3.32)	0.997	0.13 (2.98)	0.963	-0.80 (4.37)	0.854	-2.57 (3.67)	0.484	3.79 (3.73)	0.310
Household Income (ref: low)												
Medium	-0.53 (2.31)	0.817	-1.53 (2.64)	0.562	1.65 (2.37)	0.487	-0.94 (3.48)	0.787	1.80 (2.92)	0.537	-5.88 (2.96)	**0.048**
High	1.15 (2.58)	0.653	0.59 (2.95)	0.841	2.38 (2.65)	0.368	-1.20 (3.89)	0.756	3.12 (3.26)	0.338	-0.48 (3.31)	0.883
**Physical Environment**												
Park	0.12 (0.11)	0.277	0.12 (0.12)	0.315	0.12 (0.11)	0.264	0.10 (0.16)	0.547	0.30 (0.14)	**0.030**	-0.04 (0.14)	0.775
Water	-0.05 (0.41)	0.902	0.04 (0.47)	0.927	-0.23 (0.42)	0.579	-0.18 (0.62)	0.769	-0.04 (0.52)	0.927	0.35 (0.53)	0.510
Grass & Shrubbery	-0.11 (0.21)	0.584	-0.13 (0.24)	0.581	-0.09 (0.21)	0.653	0.01 (0.32)	0.961	-0.14 (0.27)	0.588	-0.29 (0.27)	0.28
Dense Vegetation	-0.11 (0.17)	0.501	-0.11 (0.20)	0.55	-0.12 (0.17)	0.49	0.02 (0.26)	0.936	-0.19 (0.22)	0.374	-0.19 (0.22)	0.38
Urbanicity (ref: urban)	2.53 (1.94)	0.192	2.76 (2.22)	0.214	2.04 (1.99)	0.305	1.13 (2.92)	0.697	2.31 (2.45)	0.345	4.86 (2.49)	0.051
Constant	82.47 (19.65)	**0.000**	80.08 (22.47)	**0.000**	89.03 (20.18)	**0.000**	66.72 (29.60)	**0.025**	87.09 (24.81)	**0.000**	86.86 (25.21)	**0.001**
R^2	0.046	0.672	0.044	0.707	0.046	0.671	0.050	0.559	0.056	0.403	0.058	0.346

Supplementary Table 6: Results of full models assessing associations between all independent variables and HRQOL indices at a 1600m buffer in the rural population

Variable	Total Scale Score		Psychosocial Health		Physical Functioning		Emotional Functioning		Social Functioning		School Functioning	
	β (SE)	*p*	β (SE)	*p*	β (SE)	*p*	β (SE)	*p*	β (SE)	*p*	β (SE)	*p*
**Intrapersonal**												
Boy (ref: girl)	-0.65 (1.30)	0.616	-0.24 (1.49)	0.869	-1.64 (1.34)	0.222	3.48 (1.97)	0.079	-1.82 (1.65)	0.271	-2.23 (1.67)	0.184
Age (years)	0.19 (0.73)	0.793	0.02 (0.83)	0.981	0.38 (0.75)	0.606	-0.09 (1.11)	0.929	-0.38 (0.93)	0.678	0.70 (0.93)	0.456
Visible Minority (ref: no)	0.72 (1.45)	0.621	1.89 (1.66)	0.256	-1.55 (1.49)	0.302	1.08 (2.20)	0.623	2.62 (1.84)	0.155	2.03 (1.87)	0.278
**Interpersonal**												
Lone Parent Household (ref: no)	0.40 (1.78)	0.820	0.71 (2.04)	0.726	-0.09 (1.83)	0.961	3.06 (2.69)	0.255	1.06 (2.25)	0.637	-2.13 (2.29)	0.354
Live in more than 1 home (ref: yes)	3.30 (2.02)	0.104	3.95 (2.31)	0.089	1.87 (2.08)	0.368	4.06 (3.05)	0.184	1.81 (2.55)	0.479	6.14 (2.60)	**0.019**
Siblings (ref: no)	1.78 (1.87)	0.340	2.46 (2.14)	0.251	0.49 (1.92)	0.798	4.24 (2.84)	0.136	5.32 (2.38)	**0.026**	-2.07 (2.40)	0.388
Mother Post-secondary (ref: no)	-1.07 (2.07)	0.604	-0.68 (2.37)	0.773	-1.45 (2.13)	0.496	-1.46 (3.12)	0.638	1.15 (2.61)	0.658	-1.86 (2.65)	0.483
Father Post-secondary (ref: no)	2.14 (1.95)	0.273	2.32 (2.22)	0.298	2.08 (2.00)	0.300	1.84 (2.94)	0.530	5.65 (2.46)	**0.022**	-0.93 (2.50)	0.710
Mother Employed (ref: no)	-2.36 (1.84)	0.201	-2.37 (2.11)	0.261	-2.24 (1.89)	0.236	-4.09 (2.77)	0.142	-1.29 (2.33)	0.579	-1.85 (2.37)	0.435
Father Employed (ref: no)	0.14 (2.90)	0.959	0.01 (3.32)	0.997	0.13 (2.98)	0.963	-0.80 (4.37)	0.854	-2.57 (3.67)	0.484	3.79 (3.73)	0.310
Household Income (ref: low)												
Medium	-0.53 (2.31)	0.817	-1.53 (2.64)	0.562	1.65 (2.37)	0.487	-0.94 (3.48)	0.787	1.80 (2.92)	0.537	-5.88 (2.96)	**0.048**
High	1.15 (2.58)	0.653	0.59 (2.95)	0.841	2.38 (2.65)	0.368	-1.20 (3.89)	0.756	3.12 (3.26)	0.338	-0.48 (3.31)	0.883
**Physical Environment**												
Park	0.15 (0.39)	0.701	0.00 (0.44)	0.991	0.43 (0.42)	0.315	-0.11 (0.59)	0.853	0.05 (0.55)	0.918	-0.08 (0.49)	0.867
Water	0.28 (0.16)	0.079	0.29 (0.18)	0.107	0.26 (0.17)	0.134	0.01 (0.24)	0.956	0.32 (0.22)	0.155	0.56 (0.20)	**0.005**
Grass & Shrubbery	0.38 (0.20)	0.063	0.40 (0.23)	0.083	0.34 (0.22)	0.128	-0.06 (0.31)	0.825	0.46 (0.28)	0.109	0.83 (0.25)	**0.001**
Dense Vegetation	0.34 (0.16)	**0.043**	0.32 (0.19)	0.088	0.37 (0.18)	**0.044**	-0.09 (0.25)	0.715	0.35 (0.23)	0.129	0.72 (0.20)	**0.001**
Region of Ontario (ref: south)	1.18 (2.12)	0.578	0.96 (2.39)	0.688	1.55 (2.31)	0.501	0.45 (3.21)	0.888	-0.18 (2.97)	0.951	2.62 (2.62)	0.319
Constant	22.75 (19.75)	0.250	13.28 (22.27)	0.551	41.21 (21.51)	0.056	61.92 (29.88)	**0.039**	-5.80 (27.64)	0.834	-19.84 (24.5)	0.419
R^2	0.0737	0.247	0.0806	0.152	0.0684	0.342	0.0851	0.108	0.1137	**0.007**	0.0971	**0.038**
